# Suberin Regulates the Production of Cellulolytic Enzymes in *Streptomyces scabiei*, the Causal Agent of Potato Common Scab

**DOI:** 10.1264/jsme2.ME15034

**Published:** 2015-09-01

**Authors:** Rebeca Padilla-Reynaud, Anne-Marie Simao-Beaunoir, Sylvain Lerat, Mark A. Bernards, Carole Beaulieu

**Affiliations:** 1 Centre SÈVE, Département de Biologie, Université de Sherbrooke, Sherbrooke (QC), J1K 2R1, Canada; 2 Department of Biology, University of Western Ontario, London (ON), N6A 5B7, Canada

**Keywords:** cellulase, glycosyl hydrolases, proteomics, *Streptomyces scabiei*, thaxtomins

## Abstract

Suberin, a major constituent of the potato periderm, is known to promote the production of thaxtomins, the key virulence factors of the common scab-causing agent *Streptomyces scabiei*. In the present study, we speculated that suberin affected the production of glycosyl hydrolases, such as cellulases, by *S. scabiei*, and demonstrated that suberin promoted glycosyl hydrolase activity when added to cellulose-, xylan-, or lichenin-containing media. Furthermore, secretome analyses revealed that the addition of suberin to a cellulose-containing medium increased the production of glycosyl hydrolases. For example, the production of 13 out of the 14 cellulases produced by *S. scabiei* in cellulose-containing medium was stimulated by the presence of suberin. In most cases, the transcription of the corresponding cellulase-encoding genes was also markedly increased when the bacterium was grown in the presence of suberin and cellulose. The level of a subtilase-like protease inhibitor was markedly decreased by the presence of suberin. We proposed a model for the onset of *S. scabiei* virulence mechanisms by both cellulose and suberin, the main degradation product of cellulose that acts as an inducer of thaxtomin biosynthetic genes, and suberin promoting the biosynthesis of secondary metabolites including thaxtomins.

*Streptomyces* species are Gram-positive bacteria that belong to the phylum *Actinobacteria* and exhibit complex morphological development. After germination, *Streptomyces* spores produce germ tubes that grow by tip extension and branch to form a vegetative mycelium. This mycelium differentiates by forming a second aerial mycelium, which eventually septates to form spores. Although most *Streptomyces* species are saprophytic soil inhabitants, some, such as *Streptomyces scabiei*, are plant pathogens (35). *S. scabiei* is one of the main agents of potato common scab. This plant pathogen induces symptoms ranging from corky patches to deep-pitted lesions on the surface of tubers, which reduce the value of affected potatoes (20). The pathogenicity of *S. scabiei* depends on the production of thaxtomins (17, 19), secondary metabolites that inhibit the biosynthesis and deposition of cellulose in plant cells (47).

The transcription of *S. scabiei* thaxtomin biosynthetic genes (*txt* genes) is induced in the presence of plant-derived materials such as suberin and cellobiose (31). The role of cellobiose in triggering the production of thaxtomins has been elucidated. Cellobiose binds the AraC/XylS transcriptional regulator, TxtR, thereby allowing the expression of *txt* genes (23). Furthermore, cellobiose has been shown to inhibit the attachment of the cellulose-utilization repressor CebR to binding sites of the *txt* gene cluster (14). On the other hand, the effects of suberin on *txt* gene expression remain unclear. Lerat *et al.*(31, 32) proposed that suberin activated the differentiation and production of secondary metabolites including thaxtomins in several *Streptomyces* species.

Suberin is a recalcitrant plant polymer composed of a polyaromatic domain covalently linked to a polyaliphatic domain (5). The polyaromatic domain, a lignin-like structure, is embedded in the primary cell wall and largely consists of the hydroxycinnamic acid, monolignol (4). The polyaliphatic domain is composed of polyesters of long-chain fatty acids and glycerol, which are located between the primary cell wall and the plasma membrane. Its chemical structure is related to cutin, a polymer covering the aerial parts of plants (39). Suberin has been suggested to play a protective role against common scab disease. A previous study reported a relationship between the phenolic acid composition of suberin and cultivar resistance to common scab (49), while Khatri *et al.*(26) observed the higher suberization of the potato periderm in a common scab-tolerant cultivar than in a susceptible one.

As soil-dwelling organisms, *Streptomyces* species feed on plant polymers. This genus is known for its ability to produce a wide variety of extra-cellular enzymes involved in the degradation of cellulose, hemicellulose, and recalcitrant plant polymers such as lignin (40, 51). *S. scabiei* has the ability to colonize the potato periderm. The outer layer of the periderm, the phellem, consists of suberized cells (26). Evidence to support *S. scabiei* at least partially degrading suberin is increasing. This bacterium was previously shown to grow on suberin as a source of carbon (29) and produced esterases in its presence (1). Furthermore, the *S. scabiei* genome encodes potential suberinase genes (29). One of these genes, *sub1* is known to be specifically induced in the presence of suberin and cutin (29). A secretome analysis of *S. scabiei* cultures grown in the presence of suberin has also been conducted in order to identify the enzymes potentially involved in the degradation of suberin (30). Several enzymes predicted to play a role in lipid metabolism were identified as candidate enzymes involved in the degradation of suberin. However, these enzymes accounted for a small fraction of the *S. scabiei* secretome, whereas glycosyl hydrolases represented the most abundant functional group of proteins in the supernatant of suberin-containing medium (30). Several cellulases have been identified among the glycosyl hydrolases; however, most *S. scabiei* strains exhibit no or poor growth on cellulose (13).

The effects of suberin on the production of glycosyl hydrolases and expression of cellulase-encoding genes were examined in the present study. Furthermore, the secretomes of *S. scabiei* grown in media containing either cellulose or both cellulose and suberin were compared. A model of the onset of *S. scabiei* virulence mechanisms by both cellulose and suberin was proposed.

## Materials and Methods

### Bacterial strain and growth conditions

*Streptomyces scabiei* strain EF-35 was isolated in Canada from a common scab lesion on a potato tuber (12). A bacterial inoculum was prepared by inoculating approximately 10^8^ spores in 25 mL of YME (4 g L^−1^ of glucose, 4 g L^−1^ of yeast extract, and 10 g L^−1^ of malt extract). The culture was incubated with shaking (250 rpm) for 48 h at 30°C, after which the bacterial cells were recovered by centrifugation (3,500×*g*) for 10 min. The bacterial inoculum was obtained by resuspending the pellet in 5 volumes of saline (NaCl 0.85%). The inoculum (200 μL) was transferred to 50 mL of a control medium (CM) composed of 0.5 g L^−1^ l-asparagine, 0.5 g L^−1^ K_2_HPO_4_, 0.2 g L^−1^ MgSO_4_·7H_2_O, 10 mg L^−1^ FeSO_4_·7H_2_O, and 0.05% (w/v) casein hydrolysate (Sigma, St-Louis, MO, USA) supplemented or not with 0.1% (w/v) suberin and/or 0.5% (w/v) of an additional carbon source: insoluble microcrystalline cellulose Avicel (EMD Millipore, Billerica, MA, USA), xylan (oat spelt, Sigma), or lichenin (also known as lichenan) (Megazyme, Wicklow, Ireland). Cultures were incubated with shaking (250 rpm) for 5 d at 30°C.

Suberin used in this study was extracted from potato tubers, as described in Kolattukudy and Agrawal (28). The amount of residual glucose in the purified polymer was determined according to Blakeney *et al.*(6). The alditol acetate derivatives of glucose, obtained by the hydrolysis, reduction, and acetylation of the suberin preparation, were quantified by gas chromatography on a Varian 3800 GC equipped with a flame-ionization detector (FID) and CP-Sil 88 column (48). The experiment was carried out with five replicates.

### Extracellular protein quantification and concentration determination

Extracellular proteins were recovered by centrifuging the bacterial cultures (3,500×*g*) for 15 min at 4°C. The protein concentrations of the *S. scabiei* culture supernatants were determined according to Bradford (7) using bovine serum albumin as a standard. In proteomic assays, supernatants were concentrated to a final volume of 500 μL using Amicon Ultra-15 Centrifugal Filters-3K, and the proteins were resuspended in 5 volumes of 100% pre-chilled acetone and kept for 3 h at −20°C. Proteins were recovered by centrifugation (14,000×*g*, 20 min, 4°C) and the protein pellets were air-dried and resuspended in 80 μL of a buffer composed of 8 M urea, 2% (w/v) CHAPS, 2% (v/v) IPG buffer pH 4–7 (GE Healthcare, Buckinghamshire, UK), 18.15 mM DTT, and 0.002% bromophenol blue stock solution in 50 mM Tris-base. A final centrifugation (14,000×*g*) was carried out for 5 min at 4°C to remove insoluble material and the protein solutions were immediately subjected to a proteomic analysis.

### Isolation of genomic DNA

Bacterial biomass was estimated by cellular DNA quantification. Genomic DNA was extracted according to the procedure of Kieser *et al.*(27). Genomic DNA was resuspended in 500 μL of a buffer containing 10 mM Tris-HCl and 1 mM disodium EDTA (pH 8.0) (TE buffer) and further purified. Two microliters of RNase (10 mg mL^−1^) was added to the DNA solution followed by an incubation for 1 h at 37°C. Chloroform (250 μL) was added to the DNA solution and mixed by inversion. The mixture was centrifuged for 10 min at 3,500×*g*. Two hundred and fifty microliters of 3 M sodium acetate, pH 5.2 and 1 mL of 2-propanol were mixed into the upper phase and the solution was centrifuged for 10 min at 3,500×*g*. The pellet was rinsed with 70% ethanol and resuspended in 400 μL of TE buffer. DNA was then quantified using a NanoDrop 2000 spectrophotometer (Thermo Scientific, Waltham, MA, USA) according to the manufacturer’s specifications.

### Enzymatic assays

Cellulase, xylanase, and licheninase activities associated with *S. scabiei* culture supernatants were determined according to Lever (33) as further described in Komeil *et al.*(30).

### Proteomics analysis

Extracellular proteins were subjected to sodium dodecyl sulfate-polyacrylamide gel electrophoresis (10% [w/v] SDS-PAGE) according to Komeil *et al.*(30). Horizontal slices were cut across the SDS-PAGE gel and used to perform a proteomic analysis. In-gel protein digestion and mass spectrometry were carried out at the Proteomics Platform of the Eastern Quebec Genomics Center (Quebec City, QC, Canada) using a quadrupole time-of-flight mass spectrometer Qq-TOF (Sciex, Concord, ON, Canada) coupled to HPLC for the separation of peptides used with a nanospray source. This system allowed for the resolution of more than 30,000 FWHM with an accuracy of <2 ppm and an acquisition rate of 50 MS/MS s^−1^.

All MS/MS spectra were analyzed for peptide identification using Mascot (Matrix Science, London, UK; version 2.2.0) to search the *S. scabiei* strain 87.22 Uniref100 database based on trypsin digestion, a fragment ion mass tolerance of 0.50 Da, and a parent ion tolerance of 2.0 Da. The search results were uploaded to the scaffold software program and a filter was set with a 99% minimum protein ID probability with a minimum number of two unique peptides in the protein, in which the cut-offs for peptide thresholds were 90%. Protein functions were reanalyzed using various databases such as GenBank, Pfam protein-domain/family, COG (50), and CAZy (34) as well as PRIAM (10) and KEGG resources (25). Phobius (24), SignalP 4.1 (43), SecretomeP (2), TatP (3), and Tatfind 1.4 (45) analyses were used to predict protein cellular localizations. A normalized spectral abundance factor (NSAF) was calculated for each protein that met the filtering criteria by dividing the proportion between the number of spectral counts (SpC) of a protein in culture medium and its molecular weight (MW) by the sum of SpC/MW of all proteins meeting the filtering criteria and found in the same medium (41).

### Gene expression

The effects of carbon sources on gene expression were determined as follows. Five milliliters of each culture medium was sampled after 5 d of growth. One milliliter of stop solution (ethanol/acidic phenol, 95:5, [v/v]) was added to each sample to prevent RNA degradation. Bacterial cells were recovered by a 10-min centrifugation at 3,500×*g* and stored at −80°C until RNA extraction. Total RNA was extracted from cells with the NucleoSpin RNA II kit (Macherey-Nagel, Düren, Germany) following the manufacturer’s instructions. Cell lysis was improved by a sonication step (2×30 s) before phenol/chloroform extraction. An additional DNA digestion step was performed after elution with the Turbo DNA-free kit (Ambion, Austin, TX, USA). cDNA was synthesized by reverse transcription using 2 μg of total RNA with the First-Strand cDNA Synthesis kit (GE Healthcare) using 72% G+C-rich random hexamers. Quantitative real-time reverse-transcription polymerase chain reaction (qRT-PCR) of gene transcripts was performed using Mx3000P (Agilent Technologies, Santa Clara, CA, USA) with the SYBR Green PCR master mix and JumpStart Taq DNA Polymerase (Sigma). PCR cycles were 95°C for 5 min, followed by 35 cycles at 95°C for 15 s and 60°C for 45 s. The primers used in this study are listed in Table 1. Gene *gyrA* was used as a reference for the internal control of relative quantification. Relative gene expression was determined using the comparative C_T_ method (44).

## Results

### Effects of the presence of suberin in polysaccharide-containing culture media on *Streptomyces scabiei* growth, extracellular protein production, and enzymatic activities

The amount of glucan remaining embedded in the suberin preparation added to the culture medium was determined by quantifying glucose concentrations. The average concentration of residual glucose was 103±8 μg mg purified suberin^−1^. The amount of glucose from suberin thus represented approximately 2% of the sugar present in control medium (CM) supplemented with cellulose (CM+C), xylan (CM+X), or lichenin (CM+L). The effects of adding suberin to the three latter media on *S. scabiei* growth, extracellular protein production, and enzymatic activity were then determined. The addition of suberin had different effects on *S. scabiei* growth depending on the polysaccharide that was available as the main carbon source. Suberin did not affect growth in xylan-, improved growth in cellulose-, and inhibited growth in lichenin-containing medium (Table 2). While suberin appeared to be a better growth substrate than cellulose for *S. scabiei*, this was not the case for either xylan or lichenin. Biomass, estimated by DNA quantification, was approximately 8 to 10-fold higher in the presence of these substrates than in CM+S (Table 2).

The addition of suberin did not modify the levels of extracellular proteins produced per μg of bacterial DNA in CM supplemented with cellulose, xylan, or lichenin. However, there were approximately 5 to 10-fold more extracellular proteins in control medium supplemented with suberin (CM+S, 141.7±11.7 μg μg of DNA^−1^) than in CM supplemented with xylan (CM+X, 28.2±2.6 μg μg of DNA^−1^) or lichenin (CM+L, 14.8±1.8 μg μg of DNA^−1^). No significant differences were observed in the amounts of extracellular proteins associated with CM+S and CM supplemented with cellulose (Table 2).

A 3- to 4-fold increase in cellulase, xylanase, or licheninase activity was observed when suberin was added to CM+C, CM+X, and CM+L media. Licheninase activity was similar in CM+L (17.4±0.8 U μg of DNA^−1^) and CM+S (19.0±4.3 U μg of DNA^−1^), whereas cellulase and xylanase activities were higher in CM+S (1.8±0.5 U μg of DNA^−1^, 35.1±8.2 U μg of DNA^−1^, respectively) than in CM+C (0.7±0.6 U μg of DNA^−1^) or CM+X (14.5±1.1 U μg of DNA^−1^), respectively.

### Effects of suberin on the *Streptomyces scabiei* secretome in cellulose-containing medium

**Fig. 1:**
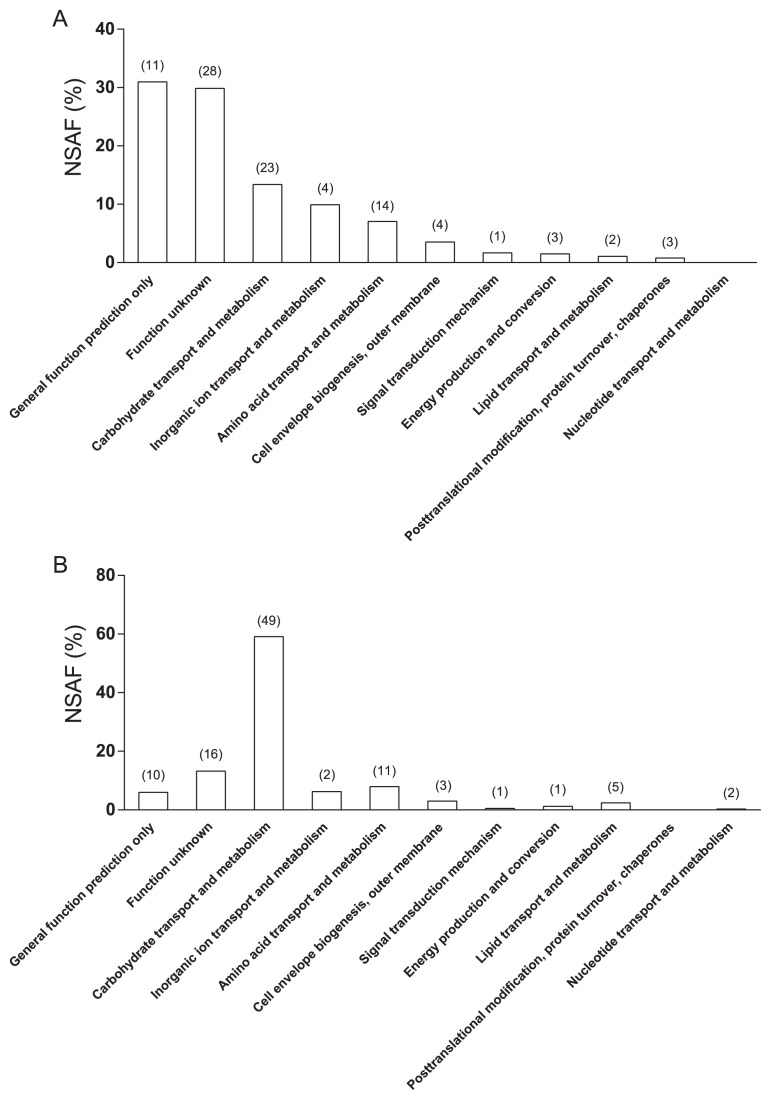
Normalized spectral abundance factor (NSAF) within functional groups of *Streptomyces scabiei* strain EF-35 secreted proteins associated with control medium supplemented with microcrystalline cellulose (A) or both microcrystalline cellulose and suberin (B). Values in brackets represent the number of proteins found within functional groups.

The addition of suberin to a cellulose containing-medium only slightly affected the diversity of the secretome. A total of 115 and 125 proteins met the filtering criteria in CM+C and in the same medium supplemented with suberin (CM+C+S), respectively (Table S1). A total of 22 and 25 proteins were predicted to have an intracellular localization in CM+C and CM+C+S, respectively. The other predicted extracellular proteins were divided into 11 functional groups. The distribution of the secreted proteins within the functional groups differed according to the growth medium. Fig. 1 shows a normalized spectral abundance factor (NSAF) and the number of proteins associated with each functional group. In CM+C, proteins associated with the “general function predicted only” group had a NSAF of 31% and included the most abundant protein in the secretome (SCAB_8801), a subtilase-like protease inhibitor with an NSAF of 26.02% (Fig. 1A and Table S1). Proteins of unknown function included 28 proteins with a NSAF of 30%. The functional group “carbohydrate transport and metabolism” was composed of 23 proteins and had a NSAF of 13% (Fig. 1A). Of these, 25 (NSAF=14.18%) were putative glycosyl hydrolases (GH) belonging to seven GH families (Table 3). The most abundant GH were the putative cellulase C9ZD50 (NSAF of 1.86%) and a putative xylanase A (C9ZE95, NSAF of 1.84%). The GH group included nine putative cellulases with a combined NSAF of 3.9% (Table 3).

The addition of suberin to CM+C resulted in a marked reduction in the abundance of the subtilase-like protease inhibitor (NSAF of 1.1%) (Table S1). In contrast, suberin led to an increase in protein abundance in the “carbohydrate transport and metabolism” group (NSAF of 59%) (Fig. 1B). The number of proteins in this group was approximatively 2-fold lower in CM+C (23 proteins) than in CM+C+S (49 proteins) (Fig. 1). In CM+C+S, the two most abundant proteins with respective NSAF of 10.3% and 9.5% were a putative xylanase A (C9ZE95) and glucose/sorbosone dehydrogenase predicted to be involved in glucose metabolism (C9Z5L1) (Table S1). When suberin was added to CM+C, the diversity of GH increased (Table 3) and these GH were distributed among 19 GH families (Table 3). Thirteen cellulases produced in CM+C+S met the filtering criteria (Table 3). These 13 predicted cellulases were more abundant in CM+C+S than in CM+C. The only cellulase that was more abundant in CM+C than in CM+C+S was cellulase B (C9Z9L5), which accounted for NSAF of 0.30% in CM+C. This cellulase B was detected in CM+C+S, but did not pass the filtering criteria.

### Effects of plant cell wall constituents on the expression of cellulase-encoding genes

**Fig. 2:**
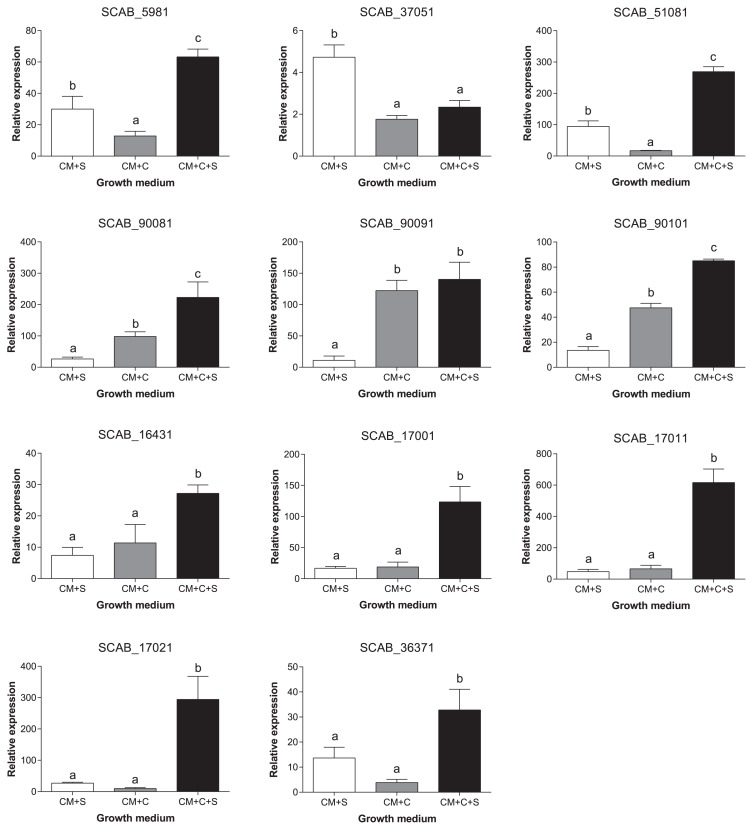
Relative expression levels (±SD) of eleven targeted cellulases found in the secretome of *Streptomyces scabiei* EF-35 grown in control medium (CM) in the presence of suberin (CM+S, white bars), microcrystalline cellulose (CM+C, gray bars), or microcrystalline cellulose supplemented with suberin (CM+C+S, black bars). Data were normalized with the *gyrA* gene used as an internal control. Data shown are representative of three replicates. Data with the same letter are not significantly different (*P*<0.05, LSD test).

The expression of 11 genes encoding cellulases found in the *S. scabiei* secretome was monitored in different growth media. The addition of cellulose, suberin or both polymers to control medium (CM) induced the gene expression of all the cellulase-encoding genes tested. The expression levels of genes encoding cellulases SCAB_16431, SCAB_17001, SCAB_17011, SCAB_17021, and SCAB_36371 were similar in media containing either suberin (CM+S) or microcrystalline cellulose (CM+C). The expression of genes encoding cellulases (SCAB_90081, SCAB_90091 and SCAB_90101) was higher in control medium supplemented with cellulose than in CM+S. In contrast, the expression of the cellulase-encoding genes SCAB_5981, SCAB_37051, and SCAB_51081 was higher in CM+S than in CM+C. When suberin was added to microcrystalline cellulose-containing medium, cellulase-encoding gene expression increased significantly, from 1.2- to 32-fold, for all genes, except SCAB_90091 and SCAB_37051 (Fig. 2).

## Discussion

A recent proteomic study (30) showed that a wide variety of extracellular glycosyl hydrolases were produced by the phytopathogen *S. scabiei* in the presence of suberin. Therefore, we speculated that suberin affects the extracellular enzyme production of *S. scabiei* when added to polysaccharides such as β-glucans (cellulose or lichenin) or xylan. The addition of suberin to glucan- or xylan-containing medium did not modify the amount of secreted proteins per μg of bacterial DNA. However, the quantity of extracellular proteins was markedly higher in suberin- and cellulose-containing media than in xylan- or lichenin-containing media. Therefore, the amount of extracellular proteins was lower in media supporting good growth (lichenin- or xylan-containing media) than in CM+S and cellulose-containing media. This upregulation in the responses of genes encoding enzymes that hydrolyze plant polysaccharides to carbon-starving conditions has been demonstrated previously (11, 53). As recalcitrant growth substrates, both cellulose and suberin may, thus, trigger the secretion of extracellular enzymes (18, 36, 37, 42). The poor ability of *S. scabiei* to grow in the presence of cellulose was unexpected considering the presence of several predicted cellulase- and β-glucanase-encoding genes in its genome. However, cellobiose, the main degradation product of cellulose, has been shown to be a poor inducer for endoglucanases in at least some streptomycetes (15), including *S. scabiei*. Although cellobiose uptake and catabolism have both been demonstrated in *S. scabiei* cells (31), *S. scabiei* cellulase activity in the supernatant of CM supplemented with cellobiose was only 0.05±0.02 U μg of DNA^−1^ (data not shown), but reached 0.7±0.6 U μg of DNA^−1^ in CM+C (see above in the Results section). The poor inducing ability of cellobiose may also explain why some streptomycetes showed higher cellulase activity on complex organic materials such as straw than on purified cellulose (15, 46, 54, 55).

The present study demonstrated that suberin was an as good as, or even a better inducer of glucanase and xylanase activities than polysaccharides. Furthermore, the addition of suberin to glucan- or xylan-containing medium increased glucanase or xylanase activity, respectively. This higher enzymatic activity may not have been due to the additional supply of polysaccharides provided by sugar residues attached to suberin because (i) the amount of glucose embedded in this lipidic polymer represented only 2% of the glucose contained in the medium, and (ii) the suberin-containing medium supernatant from the *S. scabiei* culture was unable to release reducing sugars from the suberin used in this study (data not shown).

Therefore, secretomes of *S. scabiei* cultivated in CM+C and CM+C+S were compared in order to determine if the supply of suberin induced specific glucanases or caused the general overproduction of glucanases. A proteomic analysis revealed that most GH that associated with the CM+C supernatant were predicted to cleave β-1,4 links (cellulases, xylanase, and chitinase), while several other types of GH were only found in CM+C+S medium (including pectate lyase, β-1,6 galactanase, α-N-furanosidase, α-arabinase, α-fucosidase, endo-1,3 β-d-glucosidase, and acetyl-xylan esterase). As with other soil streptomycetes, the *S. scabiei* genome encodes for a multiplicity of carbohydrate catabolic proteins, especially proteins involved in the degradation of plant-derived materials. The expression of genes coding for plant cell wall-degrading enzymes is sometimes induced by molecules that bear no structural relation to the substrate (21). For example, the production of cellulases in the absence of cellulose or its degradative products has previously been reported in *Streptomyces albaduncus* (18).

All cellulases found in CM+C were previously identified in *S. scabiei* cultures grown in the presence of suberin (30), and the amount of cellulases produced was generally higher when both polymers were present (this study). The expression of cellulase-encoding genes was also generally higher when bacteria were exposed to both substrates than to cellulose or suberin only. The presence of both cellulose and suberin had an additive or even synergistic effect on the transcription of most cellulase-encoding genes, suggesting that different environmental signals are necessary for ensuring maximal cellulase-encoding gene expression.

The stimulatory effects of suberin on cellulase-encoding gene transcription may have been due to its chemical composition. Chellapandi and Jani (9) showed that surfactants enhanced the production of endoglucanases in some *Streptomyces* isolates. Esterases that have been shown to be produced by *S. scabiei* in the presence of suberin (30, 38) presumably release long chain fatty acids with hydroxyl or epoxy moieties, which may have surfactant properties (37). Furthermore, previous studies reported that phenolic compounds, including those encountered in the aromatic fraction of suberin, regulated the production of cellulolytic enzymes in some *Streptomyces* strains (15), as well as in fungi (16, 52).

The stimulating effects of suberin on cellulase-encoding gene transcription may also have resulted from its ability to promote differentiation and secondary metabolism in *Streptomyces* species (32). Cellulase activity is low during primary metabolism in some *Streptomyces* species, but reaches a maximal level during secondary metabolism (9). However, Lerat *et al.*(31) demonstrated that cellobiose, the main degradation product of cellulose, blocked differentiation in *S. scabiei*, which exhibits a bald phenotype when grown in the presence of this disaccharide. The molecular mechanism by which cellobiose inhibits differentiation and secondary metabolism in different *Streptomyces* species currently remains unknown (31); however, the results of the present study suggest that the subtilase-like protease inhibitor encoded by SCAB_8801, the levels of which were approximately 20-fold higher in CM+C than in CM+C+S (Table S1), was involved in the phenomenon. The role of subtilase protease inhibitors in holding off differentiation has been documented in several *Streptomyces* species (8). A reduction in the amount of the subtilase-like protease inhibitor when suberin was added to a cellulose-containing medium is consistent with suberin promoting differentiation, even in the presence of cellobiose (31).

**Fig. 3:**
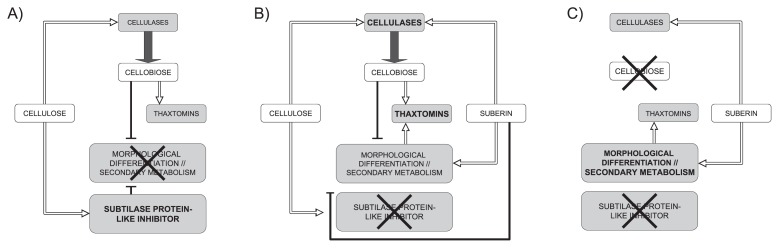
Model of the onset of *Streptomyces scabiei* virulence mechanisms by both cellulose and suberin. In the presence of cellulose only (A) or suberin only (C), the thaxtomin biosynthetic genes were only weakly expressed. In the first case, cellulases that cleave cellulose to release cellobiose were produced in low amounts. The liberated cellobiose acted as an inducer of thaxtomin biosynthetic genes. However, in the absence of an environmental signal that triggers secondary metabolism, cellobiose and/or cellulose locked *S. scabiei* in primary metabolism, possibly by allowing the production of a subtilase protease inhibitor and, thus, limiting the production of secondary metabolites such as thaxtomins,. In the presence of suberin only (C), secondary metabolism was promoted and cellulases were synthesized, whereas cellobiose, the inducer of thaxtomin biosynthetic genes was not produced due to the absence of cellulose. Thaxtomin biosynthetic genes were strongly expressed in the presence of both cellulose and suberin (B). Suberin and cellulose promoted cellulase activity and cellobiose released from cellulose induced thaxtomin biosynthetic genes. Suberin also triggered differentiation and secondary metabolism, thereby overcoming the actions of cellulose and cellobiose.

We propose the following model by which the constituents of the potato periderm promote the onset of *S. scabiei* virulence mechanisms (Fig. 3). The major periderm constituents, cellulose and suberin, both play a role in triggering the production of thaxtomins. A low amount of cellulases was produced in the presence of cellulose only (Fig. 3A), thereby allowing the release of cellobiose, the inducer of thaxtomin biosynthetic genes, which are the main *S. scabiei* virulence determinants (1, 22, 31). However, cellobiose has been shown to lock morphogenesis and secondary metabolism (31), possibly by affecting the production of a subtilase-like protease inhibitor (this work). Cellobiose, in maintaining *S. scabies* in primary metabolism, impaired the production of secondary metabolites such as thaxtomins and, consequently, only a small quantity of thaxtomins was produced. Cellulases were produced in the presence of suberin only (Fig. 3C), whereas cellobiose was not produced in the absence of accessible cellulose. Since inducers of thaxtomin biosynthetic genes are lacking, the biosynthesis of thaxtomins is low, relying only on signals promoting morphogenesis and secondary metabolism. In the presence of both suberin and cellulose (Fig. 3B), suberin may have a dual role in the onset of *S. scabiei* virulence. It may first stimulate the production of cellobiose, the transcriptional inducer of thaxtomin biosynthetic genes, by stimulating cellulase activity (this work) and the consequent release of cellobiose from cellulose. It may then inhibit the effects of cellobiose on secondary metabolism by acting as a signal molecule for morphogenesis, thereby promoting the production of secondary metabolites such as thaxtomins.

## Supplementary Information



## Figures and Tables

**Table 1:** Primers used in this study

Gene assignation	Predicted function of the corresponding protein	Primer sets (5′ to 3′)
SCAB_24291	Gyrase A (*gyrA*)	For: GGACATCCAGACGCAGTACA Rev: CTCGGTGTTGAGCTTCTCCT
SCAB_5981	Cellulase B precursor CelB	For: TGTTCAACGGGTGCCATTAC Rev: ACATAGCCGTACGAGATGCT
SCAB_16431	Cellulase CelA1	For: CATGAACCAGGCGCAGATA Rev: CCATGTAGACCCAGGTGTTG
SCAB_17001	Cellulase	For: TCGTCCAGCTGGTGATCTA Rev: GTCGATGTACTGCGTCTTGT
SCAB_17011	Cellulase	For: GAGGCGTACAGCTACCTCCTGTG Rev: GTAGAAGGAGTTGGTCGGCTGGTC
SCAB_17021	Cellulase	For: GACACCTACACCTGGAAGAAC Rev: CTTCTCCTTGCGGTTGAAGA
SCAB_36371	Xylanase/Cellulase	For: CTGAGAAGCCCGGGAAATC Rev: CACCCGTCACACACATGAA
SCAB_37051	Cellulase/Xylanase	For: ATTCTCCGGAAGCACATCAC Rev: TCCTCGAAGACCTCGTTCA
SCAB_51081	Cellulase	For: GGCATCAACTGGTTCGGTTTCGAG Rev: TGTTGTAGCCCAGCGACTTCATCT
SCAB_90081	Cellulase B precursor	For: TACAACGGCTGCCACTAC Rev: CTCGCATAGCTGTAGGAGATAC
SCAB_90091	Cellulase	For: AGGACAGCAGATACATCGAGGAGT Rev: TCAAGAAGATCGGCAACTGTGTCG
SCAB_90101	Cellulase	For: TCGAGTTGGTCGCTGAAATG Rev: AAGCGTGCCGTTGTAGTT

**Table 2:** Comparison of *Streptomyces scabiei* growth, extracellular protein concentrations, and enzymatic activities between suberin-containing control medium (100%) and control medium supplemented with a polysaccharide in the presence or absence of suberin

Medium^a^	Relative growth (%)^b^,^c^	Relative protein concentration (%)^b^,^d^	Enzymatic activity tested^b^	Enzymatic activity (%)^b^,^e^
CM+X	793	20	Xylanolytic activity	41
CM+X+S	747	20	Xylanolytic activity	126
CM+L	965	10	Licheninase activity	92
CM+L+S	472	21	Licheninase activity	387
CM+C	36	79	Cellulase activity	39
CM+C+S	105	80	Cellulase activity	170

a Control medium supplemented with xylan (CM+X), xylan and suberin (CM+X+S), lichenin (CM+L), lichenin and suberin (CM+L+S), microcrystalline cellulose (CM+C), or microcrystalline cellulose and suberin (CM+C+S).

b Data are the mean of three experiments.

c Growth was estimated by cellular DNA quantification.

d Protein concentration was estimated in μg μg of DNA^−1^.

e Enzymatic activity was estimated in U μg of DNA^−1^.

**Table 3:** Proteins involved in carbohydrate transport and metabolism were secreted into control medium supplemented with either microcrystalline cellulose or microcrystalline cellulose and suberin

UniProt accession number	Corresponding gene in the *S. scabiei* 87.22 genome	Putative function	CAZy classification	Normalized spectral abundance factor (%)	

				CM+C	CM+C+S
Cellulases					
C9YVP5	SCAB_5981	Cellulase B precursor CelB	CE1	D^a^	0.94
C9Z0D5	SCAB_8871	Cellulase	CBM13	0.12	0.38
C9ZD50	SCAB_16431	Cellulase CelA1	GH6	1.86	3.06
C9ZEP9	SCAB_17001	Cellulase	GH6, CBM2	D	1.51
C9ZEQ0	SCAB_17011	Cellulase	GH48, CBM2	0.18	1.58
C9ZEQ1	SCAB_17021	Cellulase	GH74, CBM2	ND^b^	1.47
C9YUZ2	SCAB_36371	Xylanase/cellulase	GH10, CBM2	0.19	1.77
C9YW88	SCAB_37051	Cellulase/xylanase	GH10	0.46	2.06
C9YTK2	SCAB_51081	Cellulase	GH5, CBM2	0.22	0.75
C9ZB17	SCAB_77391	Cellulose 1,4-β-cellobiosidase	NF^c^	ND	0.22
C9Z351	SCAB_86311	Cellulase	GH5	ND	0.30
C9Z9L5	SCAB_90081	Cellulase B precursor	GH12, CBM2	0.30	D
C9Z9L6	SCAB_90091	Cellulase	GH48, CBM2	0.34	2.38
C9Z9L7	SCAB_90101	Cellulase	GH6, CBM2	0.19	0.48
Other proteins involved in carbohydrate transport and metabolism					
C9ZBE6	SCAB_0631	α-l-fucosidase	GH29, CBM13	ND	0.13
C9YYV2	SCAB_3881 or SCAB_22931	Arabinofuranosidase	GH62, CBM13	ND	0.68
C9YUC5	SCAB_4961	Glucuronoarabinoxylan endo-1,4-β-xylanase	GH30	ND	0.49
C9YUG2	SCAB_5351	ABC-type sugar transport system	NF	1.27	0.71
C9YVN3	SCAB_5851	Glycosyl hydrolase	CBM32	ND	0.17
C9YVP9	SCAB_6021	Endo β-1,4-xylanase	GH10	0.25	1.37
C9YYN8	SCAB_7551	Glycosyl hydrolase	NF	0.62	D
C9Z1T6	SCAB_9291 or SCAB_91051	Lactonase	NF	ND	0.26
C9Z1U5	SCAB_9381	Exo-α-sialidase	NF	ND	0.53
C9Z507	SCAB_11431	Glycosyl hydrolase	GH43	0.22	1.11
C9Z878	SCAB_13491	Glucan endo-1,3-β-d-glucosidase	GH64	ND	0.13
C9ZD59	SCAB_16521	Arabinofuranosidase	GH43, CBM42	ND	1.81
C9ZD61	SCAB_16551	Mannosidase	GH26, CBM23	ND	0.11
C9YT63	SCAB_19561	β-fructofuranosidase	GH43, CBM13	ND	0.23
C9YUL1	SCAB_19941	Arabinofuranosidase	GH43, CBM42	0.18	0.36
C9YVX8	SCAB_21021	Xylose ABC transporter substrate-binding protein	NF	0.49	ND
C9Z5F4	SCAB_42381	ABC transporter substrate-binding protein	NF	0.26	ND
C9Z5L1	SCAB_42951	Glucose/Sorbosone dehydrogenase	NF	0.93	9.47
C9Z737	SCAB_43661	Galactan endo-1,6-β-galactosidase	GH30, CBM13	D	0.49
C9YY37	SCAB_54441	Enolase	NF	0.18	ND
C9Z2N2	SCAB_57161	Endo-β-1,6-galactanase	GH30	ND	0.21
C9Z451	SCAB_57751	Cellobiose-binding transport system associated	NF	1.46	1.72
C9ZDW4	SCAB_63891	ABC-type xylose transport-system, periplasmic	NF	1.05	0.19
C9ZFW2	SCAB_66021	β-xylosidase	GH43, CBM13	ND	0.32
C9ZFW3	SCAB_66031	Arabinofuranosidase	GH43, CBM42	D	1.09
C9YYF0	SCAB_70591	Pectate lyase	PL9	ND	0.72
C9Z2V1	SCAB_72711	Endo-1,4-β-xylanase	GH11	D	0.62
C9Z2W0	SCAB_72801	Glycosyl hydrolase	NF	ND	1.05
C9Z4J7	SCAB_74141	α-N-furanosidase	GH51	ND	0.43
C9Z623	SCAB_74681	Licheninase	NF	ND	0.44
C9ZAZ8	SCAB_77201	Glycosyl hydrolase	GH106	ND	2.33
C9ZB22	SCAB_77441	α-arabinanase	GH93, CBM13	0.13	0.24
C9ZCR4	SCAB_78891	Glycosyl hydrolase	GH30, CBM13	ND	0.17
C9ZE74	SCAB_79011	Acetyl-xylan esterase	CE2	ND	0.80
C9ZE94	SCAB_79241	Arabinofuranosidase	GH62, CBM13	0.26	1.16
C9ZE95	SCAB_79251	Xylanase A	GH10, CBM13	1.84	10.25
C9ZEC5	SCAB_79561	Glycosyl hydrolase	GHnc, CBM13	ND	0.30
C9YU29	SCAB_82021	β-mannosidase	GH5, CBM2	ND	0.51
C9Z1I5	SCAB_85231	Chitinase	GH19, CBM12	0.44	0.54
C9Z804	SCAB_89741	Cellulose-binding protein	CBM33	0.74	1.05

a D: detected. Peptides have not fulfilled the filtering criteria as described in the Materials and Methods section.

b ND: not detected.

c NF: no module has been found in the protein.
