# Supplementary material for: Suberin Regulates the Production of Cellulolytic Enzymes in Streptomyces scabiei, the Causal Agent of Potato Common Scab
Source: Microbes Environ. 2015 Sep 1;30(3):245–53. doi: 10.1264/jsme2.ME15034 (PMC4567563; doi:10.1264/jsme2.ME15034)
Supplement: Supplementary file 1 [file 30_245_s1.pdf]

**Table S1.** Proteins produced by *Streptomyces scabiei* EF-35 during growth in control medium supplemented with microcrystalline cellulose (CM+C) or control medium supplemented with microcrystalline cellulose and suberin (CM+C+S)

| Uniprot accession number | Corresponding gene in *S. scabiei* 87.22 genome | Putative function | Predicted cellular localization | Normalized spectral abundance factor (%) | |
|---|---|---|---|---|---|
| | | | | CM+C | CM+C+S |
| Amino acid transport and metabolism | | | | | |
| C9YYT5 | SCAB_8041 | Amidohydrolase | Extracellular | ND[a] | 0.25 |
| C9Z082 | SCAB_8301 | Protease | Extracellular | 0.15 | ND |
| C9Z0B6 | SCAB_8661 | Protease | Extracellular | 0.31 | ND |
| C9Z544 | SCAB_11801 | Peptidase | Extracellular | 0.24 | ND |
| C9ZGG7 | SCAB_18081 | γ-glutamyltranspeptidase | Extracellular | 0.15 | 0.47 |
| C9Z204 | SCAB_24891 | Glutamate uptake system binding subunit | Extracellular | 1.85 | 2.06 |
| C9Z5D4 | SCAB_27411 | Oligopeptide-binding transport system protein | Extracellular | 0.25 | ND |
| C9Z6U2 | SCAB_27811 | Xaa-Pro dipeptidyl-peptidase | Extracellular | 0.17 | ND |
| C9YXR8 | SCAB_37611 | Aminopeptidase | Extracellular | 0.48 | 1.98 |
| C9ZC37 | SCAB_46731 | Xaa-Pro aminopeptidase | Intracellular | 0.15 | 0.19 |
| C9ZH11 | SCAB_49881 | Muramoyltetrapeptide carboxypeptidase | Extracellular | 0.35 | ND |
| C9YTK4 | SCAB_51101 | Phosphoserine aminotransferase | Intracellular | D[b] | 0.38 |

| | | | | | |
|---|---|---|---|---|---|
| C9YXZ5 | SCAB_53981 | Peptidase | Extracellular | ND | 0.05 |
| C9ZAW6 | SCAB_62471 | Aminopeptidase | Extracellular | 0.13 | 0.78 |
| C9YTV5 | SCAB_67441 | Muramoyltetrapeptide carboxypeptidase | Extracellular | 0.27 | ND |
| C9YWP0 | SCAB_68931 | Branched-chain amino acid ABC transporter substrate-binding protein | Extracellular | 1.25 | 0.81 |
| C9YZP9 | SCAB_70761 | Solute-binding protein | Extracellular | 1.17 | 0.64 |
| C9Z1E7 | SCAB_72231 | Serine protease | Extracellular | 0.27 | 0.57 |
| C9ZCL9 | SCAB_78431 | Tripeptidylaminopeptidase | Extracellular | ND | 0.24 |
| C9Z058 | SCAB_84971 | Amidase | Extracellular | ND | 0.09 |
| Carbohydrate transport and metabolism | | | | | |
| C9ZBE6 | SCAB_0631 | α-L-fucosidase | Extracellular | ND | 0.13 |
| C9YYV2 | SCAB_3881 or SCAB_22931 | Arabinofuranosidase | Extracellular | ND | 0.68 |
| C9YUC5 | SCAB_4961 | Glucuronoarabinoxylan endo-1,4-β-xylanase | Extracellular | ND | 0.49 |
| C9YUG2 | SCAB_5351 | ABC-type sugar transport system | Extracellular | 1.27 | 0.71 |
| C9YVN3 | SCAB_5851 | Glycosyl hydrolase | Extracellular | ND | 0.17 |
| C9YVP5 | SCAB_5981 | Cellulase B precursor CelB | Extracellular | D | 0.94 |
| C9YVP9 | SCAB_6021 | Endo β-1,4-xylanase | Extracellular | 0.25 | 1.37 |
| C9Z0D5 | SCAB_8871 | Cellulase | Extracellular | 0.12 | 0.38 |

| | | | | | |
|---|---|---|---|---|---|
| C9Z1T6 | SCAB_9291 or SCAB_91051 | Lactonase | Extracellular | ND | 0.26 |
| C9Z1U5 | SCAB_9381 | Exo-α-sialidase | Extracellular | ND | 0.53 |
| C9Z507 | SCAB_11431 | Glycosyl hydrolase | Extracellular | 0.22 | 1.11 |
| C9Z878 | SCAB_13491 | Glucan endo-1,3-β-D-glucosidase | Extracellular | ND | 0.13 |
| C9ZD50 | SCAB_16431 | Cellulase CelA1 | Extracellular | 1.86 | 3.06 |
| C9ZD59 | SCAB_16521 | Arabinofuranosidase | Extracellular | ND | 1.81 |
| C9ZD61 | SCAB_16551 | Mannosidase | Extracellular | ND | 0.11 |
| C9ZEP9 | SCAB_17001 | Cellulase | Extracellular | D | 1.51 |
| C9ZEQ0 | SCAB_17011 | Cellulase | Extracellular | 0.18 | 1.58 |
| C9ZEQ1 | SCAB_17021 | Cellulase | Extracellular | ND | 1.47 |
| C9YT63 | SCAB_19561 | β-fructofuranosidase | Extracellular | ND | 0.23 |
| C9YUL1 | SCAB_19941 | Arabinofuranosidase | Extracellular | 0.18 | 0.36 |
| C9YVX8 | SCAB_21021 | Xylose ABC transporter substrate-binding protein | Extracellular | 0.49 | ND |
| C9YUZ2 | SCAB_36371 | Xylanase/cellulase | Extracellular | 0.19 | 1.77 |
| C9YUZ7 | SCAB_36421 | β-xylosidase | Intracellular | ND | 0.10 |
| C9YW88 | SCAB_37051 | Cellulase/xylanase | Extracellular | 0.46 | 2.06 |
| C9Z5F4 | SCAB_42381 | ABC transporter substrate-binding protein | Extracellular | 0.26 | ND |
| C9Z5L1 | SCAB_42951 | Glucose / Sorbosone dehydrogenase | Extracellular | 0.93 | 9.47 |

| | | | | | |
|---|---|---|---|---|---|
| C9Z737 | SCAB_43661 | Galactan endo-1.6-β-galactosidase | Extracellular | D | 0.49 |
| C9YTK2 | SCAB_51081 | Cellulase | Extracellular | 0.22 | 0.75 |
| C9YY37 | SCAB_54441 | Enolase | Intracellular | 0.18 | ND |
| C9Z2N2 | SCAB_57161 | Endo-β-1,6-galactanase | Extracellular | ND | 0.21 |
| C9Z451 | SCAB_57751 | Cellobiose-binding transport system associated | Extracellular | 1.46 | 1.72 |
| C9ZDW4 | SCAB_63891 | ABC-type xylose transport system, periplasmic | Extracellular | 1.05 | 0.19 |
| C9ZFW2 | SCAB_66021 | β-xylosidase | Extracellular | ND | 0.32 |
| C9ZFW3 | SCAB_66031 | Arabinofuranosidase | Extracellular | D | 1.09 |
| C9YYF0 | SCAB_70591 | Pectate lyase | Extracellular | ND | 0.72 |
| C9Z2V1 | SCAB_72711 | Endo-β-1,4-xylanase | Extracellular | D | 0.62 |
| C9Z2W0 | SCAB_72801 | Glycosyl hydrolase | Extracellular | ND | 1.05 |
| C9Z4J7 | SCAB_74141 | α-N-furanosidase | Extracellular | ND | 0.43 |
| C9Z623 | SCAB_74681 | Licheninase | Extracellular | ND | 0.44 |
| C9ZAZ8 | SCAB_77201 | Glycosyl hydrolase | Extracellular | ND | 2.33 |
| C9ZB17 | SCAB_77391 | Cellulose 1,4-β-cellobiosidase | Extracellular | ND | 0.22 |
| C9ZB22 | SCAB_77441 | α-arabinanase | Extracellular | 0.13 | 0.24 |
| C9ZCR4 | SCAB_78891 | Glycosyl hydrolase | Extracellular | ND | 0.17 |
| C9ZE74 | SCAB_79011 | Acetyl-xylan esterase | Extracellular | ND | 0.80 |
| C9ZE94 | SCAB_79241 | Arabinofuranosidase | Extracellular | 0.26 | 1.16 |

| | | | | | |
|---|---|---|---|---|---|
| C9ZE95 | SCAB_79251 | Xylanase A | Extracellular | 1.84 | 10.25 |
| C9ZEC5 | SCAB_79561 | Glycosyl hydrolase | Extracellular | ND | 0.30 |
| C9ZFY5 | SCAB_79861 | Xylose isomerase | Intracellular | ND | 0.70 |
| C9YU29 | SCAB_82021 | β-mannosidase | Extracellular | ND | 0.51 |
| C9Z1I5 | SCAB_85231 | Chitinase | Extracellular | 0.44 | 0.54 |
| C9Z351 | SCAB_86311 | Cellulase | Extracellular | ND | 0.30 |
| C9Z804 | SCAB_89741 | Cellulose-binding protein | Extracellular | 0.74 | 1.05 |
| C9Z9L5 | SCAB_90081 | Cellulase β precusor | Extracellular | 0.30 | D |
| C9Z9L6 | SCAB_90091 | Cellulase | Extracellular | 0.34 | 2.38 |
| C9Z9L7 | SCAB_90101 | Cellulase | Extracellular | 0.19 | 0.48 |
| Cell envelope biogenesis, outer membrane | | | | | |
| C9Z1V1 | SCAB_9441 | Sugar isomerase | Intracellular | ND | 0.08 |
| C9Z234 | SCAB_25191 | 4-hydroxy-tetrahydrodipicolinate synthase | Intracellular | ND | 0.38 |
| C9YT92 | SCAB_34981 | Lipoprotein | Extracellular | 1.71 | 2.24 |
| C9YUU8 | SCAB_35931 | Glycosyl hydrolase | Extracellular | 0.37 | ND |
| C9YXT6 | SCAB_37811 | Membrane protein | Extracellular | 0.97 | ND |
| C9Z8V2 | SCAB_45141 | D-alanyl-D-alanine carboxypeptidase | Extracellular | ND | 0.45 |
| C9YWP7 | SCAB_69011 | Lytic transglycosylase | Extracellular | 0.45 | 0.34 |

| | | | | | |
|---|---|---|---|---|---|
| Energy production and conversion | | | | | |
| C9Z8E8 | SCAB_28761 | ATP synthetase β subunit | Intracellular | 0.49 | 0.22 |
| C9Z8F0 | SCAB_28781 | ATP synthase α subunit | Intracellular | 0.51 | 0.14 |
| C9YTG2 | SCAB_35681 | Malate dehydrogenase | Intracellular | 0.91 | 0.62 |
| C9YTR7 | SCAB_67061 | Dihydrolipoyl dehydrogenase | Intracellular | 3.38 | 3.91 |
| C9YTU0 | SCAB_67291 | Cytochrome C oxidase subunit II | Extracellular | 0.44 | ND |
| C9YTU6 | SCAB_67351 | Cytochrome C heme-binding subunit | Extracellular | 0.83 | ND |
| C9YVE7 | SCAB_68221 | Nitrate reductase | Extracellular | 0.24 | ND |
| C9YY62 | SCAB_69701 | Glyceraldehyde-3-phosphate dehydrogenase | Intracellular | ND | 0.14 |
| C9ZE86 | SCAB_79151 | Cytokinin dehydrogenase | Extracellular | ND | 1.20 |
| Unknown function | | | | | |
| C9YUA5 | SCAB_4761 | | Extracellular | 0.70 | D |
| C9YVL7 | SCAB_5681 | | Extracellular | ND | 0.40 |
| C9YX78 | SCAB_6661 | | Extracellular | 0.78 | ND |
| C9YYN8 | SCAB_7551 | | Extracellular | 0.62 | D |
| C9Z516 | SCAB_11521 | | Extracellular | 0.76 | ND |
| C9Z6Q2 | SCAB_12841 | | Extracellular | ND | 0.22 |
| C9ZGH2 | SCAB_18141 | | Intracellular | ND | 0.32 |

| | | | | |
|---|---|---|---|---|
| C9YVU0 | SCAB_20641 | Extracellular | ND | 0.20 |
| C9Z3S4 | SCAB_26361 | Extracellular | 1.31 | ND |
| C9Z704 | SCAB_28451 | Extracellular | 0.78 | ND |
| C9ZBP7 | SCAB_30501 | Intracellular | 0.20 | ND |
| C9ZF64 | SCAB_33511 | Intracellular | 0.59 | ND |
| C9ZGQ1 | SCAB_33981 | Extracellular | 2.41 | 2.48 |
| C9YTD0 | SCAB_35361 | Extracellular | 0.75 | 1.51 |
| C9YTG1 | SCAB_35671 | Extracellular | 0.29 | ND |
| C9YUT0 | SCAB_35731 | Extracellular | 1.17 | 1.44 |
| C9YW90 | SCAB_37071 | Extracellular | 4.45 | ND |
| C9YZD0 | SCAB_39131 | Extracellular | 0.65 | ND |
| C9Z0Q1 | SCAB_39341 | Intracellular | 0.13 | D |
| C9Z0X0 | SCAB_40041 | Extracellular | 1.04 | D |
| C9Z759 | SCAB_43891 | Extracellular | 1.10 | 0.45 |
| C9Z765 | SCAB_43951 | Extracellular | D | 0.38 |
| C9Z7A3 | SCAB_44341 | Extracellular | 0.78 | 0.27 |
| C9Z8P6 | SCAB_44591 | Extracellular | 0.27 | ND |
| C9ZC25 | SCAB_46611 | Extracellular | 1.27 | ND |
| C9ZC26 | SCAB_46621 | Extracellular | 0.45 | ND |
| C9YV41 | SCAB_52001 | Extracellular | 1.17 | 1.24 |

| | | | | | |
|---|---|---|---|---|---|
| C9YY35 | SCAB_54421 | | Extracellular | 0.55 | ND |
| C9Z2I9 | SCAB_56741 | | Extracellular | 0.83 | ND |
| C9ZDY2 | SCAB_64081 | | Extracellular | 2.78 | 1.72 |
| C9ZHF3 | SCAB_66761 | | Extracellular | 1.09 | ND |
| C9YTT6 | SCAB_67251 | | Extracellular | 0.17 | 0.28 |
| C9Z1G8 | SCAB_72441 | | Extracellular | ND | 0.17 |
| C9Z4J0 | SCAB_74081 | | Extracellular | ND | 1.06 |
| C9Z4K8 | SCAB_74261 | | Extracellular | 1.34 | ND |
| C9Z9C8 | SCAB_76271 | | Intracellular | 0.95 | 0.44 |
| C9YU74 | SCAB_82491 | | Extracellular | 0.17 | ND |
| C9Z7Y9 | SCAB_89571 | | Extracellular | 0.47 | ND |
| C9Z9T4 | SCAB_90811 | | Extracellular | 1.70 | 0.79 |
| C9Z9U4 | SCAB_90901 | | Extracellular | D | 0.64 |
| General function prediction only | | | | | |
| C9Z0C3 | SCAB_8741 | ABC transporter substrate-binding protein | Extracellular | 0.18 | ND |
| C9Z0C9 | SCAB_8801 | Subtilase-type protease inhibitor | Extracellular | 26.02 | 1.15 |
| C9Z3C3 | SCAB_10031 | Cucumisin | Extracellular | 0.44 | ND |
| C9Z862 | SCAB_13321 | X-prolyl-dipeptidyl aminopeptidase | Extracellular | ND | 0.30 |
| C9Z871 | SCAB_13411 | Oxidoreductase | Extracellular | D | 0.57 |

| | | | | | |
|---|---|---|---|---|---|
| C9YVV0 | SCAB_20741 | Superoxide dismutase | Extracellular | 0.53 | ND |
| C9ZF82 | SCAB_33691 | Transglycosylase domain-containing protein | Extracellular | 1.42 | D |
| C9ZGS4 | SCAB_34211 | Galactose oxidase | Extracellular | ND | 0.09 |
| C9ZGU9 | SCAB_34471 | Transferase lipoprotein | Extracellular | 0.27 | ND |
| C9Z5M7 | SCAB_43121 | Serine/threonine protein kinase | Extracellular | 0.08 | 0.10 |
| C9Z760 | SCAB_43901 | Secreted hydrolase | Extracellular | 0.79 | 0.72 |
| C9Z770 | SCAB_44001 | Protein-lysine 6-oxidase | Extracellular | 0.26 | ND |
| C9ZAI3 | SCAB_45561 | Serine/threonine protein kinase | Extracellular | 0.36 | D |
| C9ZAN4 | SCAB_46111 | Single-stranded DNA-binding protein | Extracellular | D | 0.61 |
| C9Z2V8 | SCAB_72781 | Penicillin acylase | Extracellular | ND | 0.44 |
| C9ZE96 | SCAB_79261 | Feruloyl esterase | Extracellular | 0.63 | 1.88 |
| C9YU69 | SCAB_82441 | Feruloyl esterase | Extracellular | ND | 0.13 |
| Inorganic ion transport and metabolism | | | | | |
| C9YUK3 | SCAB_19841 | Aliphatic sulfonate ABC transporter substrate-binding protein | Extracellular | 1.80 | 3.50 |
| C9ZFJ5 | SCAB_49311 | High-affinity phosphate-binding protein | Extracellular | 6.65 | 2.76 |
| C9Z473 | SCAB_57981 | Protein DesF, iron transport system | Extracellular | 0.38 | D |
| C9Z7C8 | SCAB_59731 | Superoxide dismutase | Intracellular | D | 0.28 |
| C9ZAS1 | SCAB_61981 | ABC-transporter metal-binding | Extracellular | 1.10 | ND |

| | | | | | |
|---|---|---|---|---|---|
| | | lipoprotein | | | |
| C9YTX8 | SCAB_67681 | Bacterioferritin | Intracellular | ND | 0.35 |
| C9YVE4 | SCAB_68191 | Alkaline phosphatase | Intracellular | ND | 0.18 |
| Lipid transport and metabolism | | | | | |
| C9ZG71 | SCAB_3021 | Triacylglycerol lipase | Extracellular | 0.67 | 0.55 |
| C9Z6Y6 | SCAB_28271 | Cholesterol esterase | Extracellular | 0.41 | 0.75 |
| C9YTK3 | SCAB_51091 | Esterase-lipase | Extracellular | ND | 0.57 |
| C9YY49 | SCAB_54571 | Acetyl-CoA C-acyltransferase | Intracellular | ND | 0.16 |
| C9Z5Z2 | SCAB_74351 | Glycerophosphoryl diester phosphodiesterase | Extracellular | D | 0.36 |
| C9ZCR0 | SCAB_78851 | Sphingolipid ceramide N-deacylase | Extracellular | ND | 0.15 |
| Nucleotide transport and metabolism | | | | | |
| C9ZGX4 | SCAB_49491 | 5'-nucleotidase | Extracellular | ND | 0.21 |
| C9Z7F8 | SCAB_60011 | Nucleoside diphosphate kinase | Intracellular | D | 1.12 |
| C9YVK8 | SCAB_68841 | 5'-nucleotidase | Extracellular | ND | 0.12 |
| | | Posttranslational modification, protein turnover, chaperones | | | |
| C9Z578 | SCAB_26841 | Serine protease | Extracellular | 0.35 | ND |
| C9YUY5 | SCAB_36301 | 10 kDa chaperonin | Intracellular | 2.00 | ND |
| C9Z5G9 | SCAB_42541 | Chaperone protein DnaK | Intracellular | 0.21 | D |

| | | | | | |
|---|---|---|---|---|---|
| C9ZC44 | SCAB_46811 | Protease | Extracellular | 0.28 | ND |
| C9ZH64 | SCAB_50441 | 60 kDa chaperonin | Intracellular | 0.28 | D |
| C9Z989 | SCAB_61561 | Chaperone protein DnaJ | Intracellular | 0.15 | ND |
| C9ZAQ3 | SCAB_61791 | Protein-arginine deiminase | Extracellular | 0.14 | ND |
| Morphological differentiation | | | | | |
| C9ZD97 | SCAB_31531 | BldKB-like transport system extracellular solute-binding protein | Intracellular | 0.29 | ND |
| Signal transduction mechanism | | | | | |
| C9Z0L9 | SCAB_24621 | TerD-like stress protein | Extracellular | D | 0.51 |
| C9YXX5 | SCAB_38231 | Cold shock protein | Extracellular | 1.67 | ND |
| C9ZH47 | SCAB_50261 | TerD-like stress protein | Intracellular | 0.50 | ND |
| C9ZE07 | SCAB_64331 | TerD-like stress protein | Intracellular | 0.80 | 0.34 |
| C9ZHS9 | SCAB_81661 | TerD-like stress protein | Intracellular | 1.90 | 0.76 |
| Coenzyme metabolism | | | | | |
| C9Z1Y4 | SCAB_9771 | Cobalamin biosynthesis protein cobN | Intracellular | ND | 0.26 |
| Defense mechanism and virulence | | | | | |
| C9ZAA4 | SCAB_29931 | Superoxide dismutase | Intracellular | D | 0.45 |

| | | | | | |
|---|---|---|---|---|---|
| C9Z785 | SCAB_44161 | β-lactamase | Intracellular | 0.17 | 0.68 |
| Translational, ribosomal structure and biogenesis | | | | | |
| C9Z240 | SCAB_25251 | Polyribonucleotide nucleotidyltransferase | Intracellular | 0.25 | 0.34 |
| C9Z3N7 | SCAB_25991 | Ribosome-recycling factor | Intracellular | 0.76 | ND |
| C9YW92 | SCAB_37091 | Elongation factor Tu | Intracellular | 0.09 | ND |
| C9YWQ2 | SCAB_69061 | 30S ribosomal protein S1 | Intracellular | D | 0.12 |

[a] ND: not detected.

[b] D: detected. Peptides have not fulfilled the filtering criteria described in the Materials and Methods section.
